# The surveillance state of behavioral automation

**DOI:** 10.1016/j.conb.2011.11.004

**Published:** 2012-02

**Authors:** Andreas T Schaefer, Adam Claridge-Chang

**Affiliations:** 1 Behavioural Neurophysiology, Max-Planck-Institute for Medical Research, Jahnstr. 29, 69120 Heidelberg, Germany; 2 Laboratory of Neurogenetics, Neuroscience Research Partnership, Agency for Science, Technology and Research, Singapore 138673, Singapore; 3 Duke-NUS Graduate Medical School, Singapore 169857, Singapore; 4 Wellcome Trust Centre for Human Genetics, University of Oxford, Oxford OX3 7BN, United Kingdom

## Abstract

Genetics’ demand for increased throughput is driving automatization of behavior analysis far beyond experimental workhorses like circadian monitors and the operant conditioning box. However, the new automation is not just faster: it is also allowing new kinds of experiments, many of which erase the boundaries of the traditional neuroscience disciplines (psychology, ethology and physiology) while producing insight into problems that were otherwise opaque. Ironically, a central theme of current automatization is to improve observation of animals in increasingly naturalistic environments. This is not just a return to 19th century priorities: the new observational methods provide unprecedented quantitation of actions and ever-closer integration with experimentation.

## Highlights

► Behavioral automation is accelerating experimental throughput. ► Automation is integrating behavior with physiology. ► Comprehensive observation allows quantitative description of complex behaviors. ► Observational methods are being integrated with psychology experiments.

## Introduction

The awe-inspiring rise of genomics was made possible by the automatization of DNA sequencing by molecular biologists, engineers and computer scientists working together. Knowledge of genomes in turn has aided the production of rapidly expanding collections of transgenic animal strains. However, while genomics and genetics have greatly expanded, there has been — until recently — no comparable expansion in our capacity to functionally characterize the brains of mutant animals. Since the meaning of a brain is the behavior that it produces, the field has thus begun to increase the automation of behavioral assays. The same kinds of teams seen in genomics are tackling this problem: biologists, engineers, computer scientists and polymaths working to automate and digitize animal behavior experiments. Here, we will assess the implications of such automatization and digitization on the types of behavioral experiments performed as well as on the kind of data that can be obtained. While these technologies are having a great impact across all behavior science, we will primarily focus on the mainstream neurogenetic systems — mice and flies — with some mention of worms and fish.

## Automation increases throughput

Current automatization efforts are achieving their primary goals by increasing experimental throughput and accelerating the phenotyping process. Automation has been important from the beginning of modern neurogenetics with the use of activity monitors to screen for circadian mutants in *Drosophila*; these techniques continue to be relevant today. One example, a drug screen using high-throughput activity monitoring in zebrafish found hundreds of drugs that influence rest/wake states [1]. In another example, the availability of cheap webcams inspired the development of pySolo, an open source tool that captures and analyses data from hundreds of flies simultaneously and does so with better spatial resolution than conventional monitors [2].

Social behaviors are a great target for automatization, as they often comprised complex component actions that require time-consuming eye scoring from video, making them very low-throughput. In *Drosophila* aggression, there are at least seven action-types between two aggressive flies, including a wing threat stance, lunging and boxing; identification of such actions may require information about limb position (e.g. wing threat) or the stance of the participants (e.g. boxing, where the flies rear up). To make these behaviors accessible to high-throughput screening the CADABRA software was developed that is able to locate two flies’ bodies, heads and wings from video [3]. From the tracked body parts, geometrical features such as velocity or wing angle are computed and used for action classification, thus producing ethograms for both flies. This process decreases the time required to produce such ethograms from video by roughly 1000-fold and also allows the detection of subtle differences between strains that previously would have cost prohibitive time.

## Automation allows new kinds of physiology experiments

While combining physiology with behavior has been done in larger animals for decades, automation is allowing this method to be used in the smaller neurogenetic animals and in new ways.

*Drosophila*'s minute size has been a challenge to physiologists, but recently two groups described fixed-head physiology preps for behaving flies. Both rigs incorporate virtual reality screens, though one examines flight behavior with electrophysiology [4^•^] while the other accommodates a fly walking on a ball with optophysiology [5]. Both groups were able to use this method to show that the responsiveness of motion-sensitive visual neurons was increased when animals became active [6^•^]. That locomotion has such a profound impact on sensory dynamics suggests that no part of the brain is immune to the effects of activity and that non-behaving preparations will always be second best.

In worms, a motorized stage was used with feedback to re-center a freely moving animal, allowing continuous recording of ratiometric fluorescent signal [7]. In a second system, automated tracking was combined with an LCD projector for optogenetic physiological control of neural activity [8]. Since projectors have several color channels, neural activation and inactivation could be actuated simultaneously. For both systems, the integration of optical systems with tracking and transgenic interventions meant that single-neuron specificity in freely moving animals was achieved.

Similarly, in flies we see behavioral automation methods used in combination with transgenic and physiological manipulations. The T-maze for olfactory conditioning was automatized to track individual flies during learning while controlling odor and shock stimulus inputs. This allowed action-contingent laser targeting for optogenetic intervention in freely behaving animals, permitting the determination of the dopaminergic neural cluster sufficient for memory formation [9^•^].

In rodents we see two kinds of preparations emerge: conditioning paradigms for head-fixed preparations that use licking or lever pressing as the behavioral output (reviewed in [10]), and new virtual environments that permit the subject to engage in locomotor behavior [11]. In both cases, acquiring high temporal resolution behavioral data simultaneously and time-locked to physiological recordings or interventions greatly strengthens arguments for causality.

## Automation promises to improve reproducibility

Most genetic modifications have pleiotropic effects, so the meaningful interpretation of any one behavior often critically depends on knowledge of other phenotypes. For example, to make a claim about specific cognitive abnormalities, one would have to know that basic sensorimotor functions are intact. Responding to this concern, test batteries were developed where mice were systematically channeled through extensive tests of general health, reflexes, motor function and cognitive abilities (reviewed in [12]).

However, between-laboratory standardization has proven difficult, with seemingly uncontrollable idiosyncrasies within each laboratory or even for each experimenters contributing to systematic errors [13,14]. One possible mitigation is to heterogenize methodology at each site; however, the resulting variability is the natural enemy of smaller (genetic or other) modifications where behavioral phenotypes might be more subtle yet very informative [15]. A second mitigation would be to reduce or even remove animal handling altogether. For example, continuous versions of the (previously handling-intensive) T-maze spatial memory test have been introduced; rodents perform learning tasks for hours without intervention [16–18]. Importantly, careful validation of automated versions is necessary to ensure that the automated version of a paradigm is indeed probing the same behavior [19]. Further automatization that obviates human contact might further decrease variability by removing handling effects, thus allowing for virtually complete standardization between laboratories. Direct systematic comparisons [13] are necessary to probe this important aspect.

## Automated observation: the rodent home cage

For much of the twentieth century, animal behavior studies fell into two schools: the psychologists who emphasized elaborate laboratory experimentation and the ethologists who emphasized detailed field observation. Classical ethology aimed to identify behavioral patterns in the natural situations in which they are executed, asking the question: ‘What *does* the brain do?’ Automation is allowing neurogeneticists to make richly detailed, ethology-inspired descriptions of behavior. We see this theme in all the neurogenetic systems, though implemented in different ways.

In mice, the inspection of home cage behavior through videotaping has proven a sensitive discriminator of strain differences and disease phenotypes, scoring actions such as walking, grooming, climbing and sleeping. However, the time-intensiveness and the subjectivity of scoring by eye have limited the widespread use of these techniques. Commercial systems such as EthoVision or ANY-maze [20] can track simple movement patterns, but now attempts are being made to entirely replace the human scorer. Using the commercial HomeCageScan system, Steele and co-workers detected posture and movement, quantified rest and awake spaces, grooming, sniffing, rearing and jumping and used the extracted features to reveal key features of several disease models [21^•^]. Recently developed open source software further improves the quality and availability of this analysis methodology [22].

One disadvantage to video tracking is the requirement for unobstructed images [23], thus, for example, precluding environmental enrichment of the home cage. Alternatives are systems that detect floor movements (e.g. LABORAS) that have fewer constraints on the complexity of the environment [24]. Finally, Goulding *et al*. used conventional photo-beams, lickometers and weight platforms to detect eating, drinking and general movement [25^•^]. Despite the seemingly limited detectable repertoire, a combination of high temporal resolution, comprehensiveness and superb data analysis allowed the authors to define distinct behavioral patterns and discriminate changes robustly associated with an early onset obesity phenotype.

## Automated observation: multiple animals

The rodent home cage methods described above are largely restricted to individual animals, a limitation that introduces two issues: social deprivation itself produces abnormalities, and having to prepare many single-animal home cages is slow. In response, radio-frequency identification (RFID) methods are being used to unambiguously track individuals in group cages (e.g. the IntelliCage system [26]). Strategic placement of detection coils next to feeders, bridges, scales and more allows the reconstruction of movement patterns, place preferences, weight development and even social interactions [27^•^]. Recent applications demonstrated its use for long-term observations in the development of neurodegenerative disease for animals both in standard home cage [28] and semi-natural environments [27^•^]. While these methods are relatively coarse, combination with video tracking and new data analysis methods promise to open further possibilities for sensitive phenotyping.

**Figure 1:**
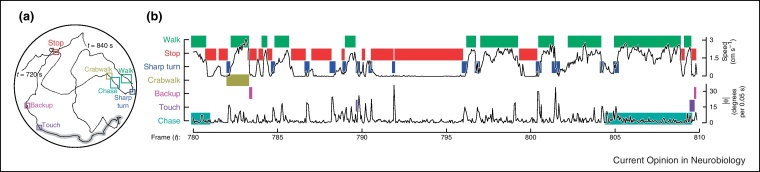
A system for tracking multiple animals and classifying behaviors: ctrax. **(a)** A representative trace of 1 of 20 simultaneously tracked flies from a 2 min interval. The trace is annotated with instances of seven automatically classified behaviors. **(b)** A time-resolved ethogram of 30 s, showing the behavioral epochs and two locomotor measures, speed and turning angle. Images from [29^••^] with permission.

While video tracking of multiple animals with identity maintenance remains largely inaccessible in rodents, it was recently achieved for *Drosophila*. Ctrax is an open source machine vision tool that tracks multiple walking flies while maintaining identity for extended intervals [29^••^]. Each animal's location and orientation on a surface is determined, and this tracking data is then classified into a variety of locomotor and interactive behaviors with a human-trained machine-learning algorithm to produce richly annotated timelines (‘automatic ethograms’). In addition to increasing throughput and furnishing rich qualitative detail, this method also quantifies behavior in ways previously only accessible in single-fly assays. Complementing this approach, an arena was designed to limit flies to a monolayer, using a gradual incline to spread out wall-walkers and using a hydrophobic coating to prevent ceiling-walking [30]. While this system is clearly not entirely naturalistic, the Ctrax tracking and classification system yields observational information about ‘default’ behaviors that has no eye-scored predecessor, perhaps due to the laborious nature of such a task (Figure 1).

Another extravagant technical achievement in behavioral observation technology is a multi-camera system that is capable of tracking of multiple flies in flight, in real-time [31]. The system was used in conjunction with a virtual reality flight arena with video input delivered to the floor and two walls. This method enabled an analysis of the combination of visual reflexes that flies use to control altitude [32]. As with Ctrax, these flying-fly trackers have the explicit aim of improving behavioral quantitation in increasingly naturalistic environments.

## Automation enables new kinds of quantitative analysis

The above examples show how automated observation can help classify behaviors, quantify their frequency and thereby increase throughput [21^•^,25^•^]. However it is also clear that automation is aiding more fine-grained analyses of animal motors. Recent work in worms illustrates these aspects. Several recent papers describe tracking multiple animals at postural resolution. One method emphasizes environmental control, using a structured environment to simulate soil, and microfluidic delivery of precise odor streams, pulses and gradients [33]. This method gives fine-grained information about how odors drive motor programs in wild type and mutant animals. Beyond detecting the seven known classified locomotor patterns, this system also identified three new behaviors. A second method implements simultaneous real-time tracking of hundreds of animals on conventional Petri dishes to facilitate high-throughput phenotyping [34]. A pilot screen for tap habituation defects revealed several mutants with abnormal habituation, demonstrating how this high-throughput method will expand the genome–phenome matrix. This study also confirmed a third study that found that much of worm locomotion can be summarized as transitions between four ‘eigenworms’, postures that correspond to the principle components of body curve geometry [35^••^]. These four body-shape attractors may indeed be underpinned by four corresponding pattern generator states. This result and similar findings in other systems raise hope that low-dimensional descriptions of complex behaviors will refine or even supplant named classification and may guide the search for underlying circuits. Such a fine-grained analysis of behavior might even lead to a truly comprehensive description of a species’ behavior in terms of its muscle contraction patterns.

## Automation improves psychology experiments

Psychology challenges animals with tasks that probe the limits of sensory discrimination and memory, asking the question: ‘What *can* the brain do?’ Automation has long benefited psychology [36–38]. In flies, there are increasing efforts to develop assays of important cognitive functions. To study externally induced arousal, an air puff device was developed to mechanically startle flies into an acute state of elevated activity. Multiple-animal tracking was used to assess walking speed, which was subsequently analyzed for a variety of metrics. This paradigm was used to screen for lines that displayed aberrant arousal behavior, discovering a dopamine receptor mutation that increased acute arousal but decreased night-time activity. Selective recovery of the dopamine receptor was used to show a neuroanatomical delineation between environmentally induced acute arousal and nocturnal wakefulness. Inspired by the Morris water maze, two groups used video tracking and localized heat control to establish assays of place learning in flies [39,40]. Using arenas tiled with thermoelectric devices, a cool patch could be allocated on an otherwise painfully hot floor, acting to substitute for the water maze's shallow reef. It was shown that place learning was dependent on visual cues and a screen of candidate brain regions found that a specific neural cluster was required for this novel form of learning [40].

In rodents, there are now myriad modern versions of the operant training box. Using millisecond detection of an animal's responses, some of these test the limits of cognition and sensation [41–45], dissecting subtly different cognitive abilities [45–47]. Other systems use touch-screens to capitalize on a mouse's tendency to nose-poke [48,49] thereby incorporating the operandum into a versatile stimulus presentation device. Furthermore, this allows animals with reduced movement to successfully perform cognitive tasks [50]. For aversive responses, accelerometers can be used to give temporally precise reports of startle and freezing behavior (e.g. [51], commercial systems include the startle response system from San Diego Instruments).

## Automation affords combining naturalistic observation with experimentation

**Figure 2:**
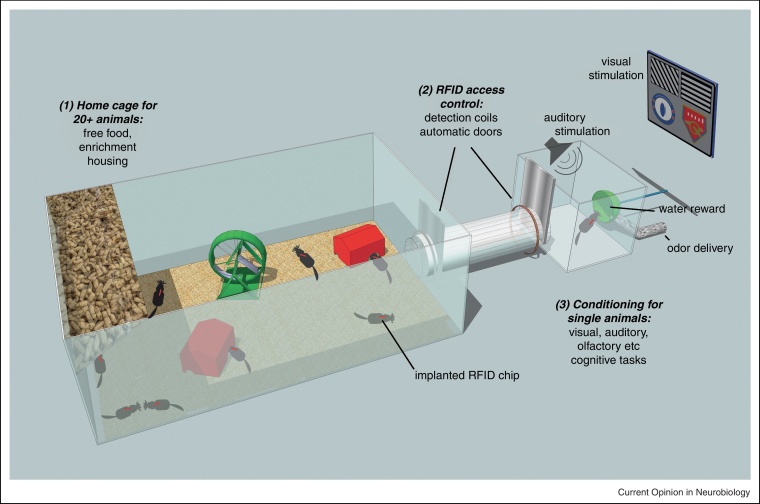
Tagging individual mice with RFID chips allows for social housing yet through distinct gates and tunnels makes it possible to automatically separate and channel individual mice to arbitrary conditioning units [26,27^•^,54^•^]. There, standard automatic high-resolution behavioral analysis can take place using defined auditory, visual, olfactory, somatosensory or other stimuli (Schaefer & Bus, Soc Neurosci. Abstr. 670.5 2010). This approach combines interruption free group housing over periods of months with highly quantitative and sensitive behavioral analysis in a low maintenance, high-throughput and cost effective way.

Automated conditioning of mouse behavior remains a time consuming process, made worse by the need for repeated transfers to and from the home cage. One solution is to leave animals inside the conditioning box for extended periods of time [16], but this is an unrealistically expensive solution for large cohorts of mice. Thus, attempts have been made to integrate training with a social home cage and transponder tracking. In the IntelliCage system, basic experiments such as place learning, novel object recognition, passive avoidance, among others are possible using transponder-keyed barriers [26,52,53]. Others have introduced RFID-controlled tunnels and gates that more generally control movement from a large group cage to conditioning boxes for individual animals ([54^•^]; ‘AutomatedGroupCage’ [PhenoSys]; ‘AnimalGate’ [NewBehavior]). Furthermore, custom-designed systems are being developed for tailored high precision conditioning (Schaefer & Bus, Soc Neurosci. Abstr. 670.5 2010). All these approaches bring together prolonged animal home cage observation with tightly controlled conditioning paradigms, allowing us to efficiently and sensitively probe cognitive functions in large cohorts of socially housed animals (Figure 2).

## Conclusion

Automation is accelerating experimental throughput and having diverse effects on how behavioral neuroscience is done. It is transforming physiology by maintaining animals in an active mode, facilitating quantitative analysis of motor patterns, making new psychology experiments accessible and integrating ethology-type observation with psychology experiments. Two developments are particularly striking. The first is the tracking and sorting of socially housed experimental animals that permits efficient execution and contextual interpretation of detailed conditioning experiments. The second is high-resolution motion capture methods that provide fodder to analytical tools for automated classification and dimensionality reduction. In these developments and throughout the literature we see the central theme as being improved observational methods in increasingly naturalistic conditions.

In the future, sensitive ‘panopticon’ systems will capture joint-resolved data from entire colonies inhabiting complex naturalistic environments over life spans. The panopticon colony will be the subject of study, with telemetric physiology and automated psychology experiments integrated as a part of the social group's existence. Scientists well versed in data analysis will interpret the enormous data sets produced by these systems to produce more quantitative, impartial and broader insights into what brains do.

## References and recommended reading

Papers of particular interest, published within the period of review, have been highlighted as:

• of special interest
•• of outstanding interest
